# Mechanisms Underlying Anti-Inflammatory and Anti-Cancer Properties of Stretching—A Review

**DOI:** 10.3390/ijms231710127

**Published:** 2022-09-04

**Authors:** Małgorzata Król, Patrycja Kupnicka, Mateusz Bosiacki, Dariusz Chlubek

**Affiliations:** 1 Department of Biochemistry and Medical Chemistry, Pomeranian Medical University, Powstańców Wlkp. 72, 70-111 Szczecin, Poland; 2 Chair and Department of Functional Diagnostics and Physical Medicine, Pomeranian Medical University, Żołnierska 54, 71-210 Szczecin, Poland

**Keywords:** stretching, inflammation, collagen, cytokines, cancer

## Abstract

Stretching is one of the popular elements in physiotherapy and rehabilitation. When correctly guided, it can help minimize or slow down the disabling effects of chronic health conditions. Most likely, the benefits are associated with reducing inflammation; recent studies demonstrate that this effect from stretching is not just systemic but also local. In this review, we present the current body of knowledge on the anti-inflammatory properties of stretching at a molecular level. A total of 22 papers, focusing on anti-inflammatory and anti-cancer properties of stretching, have been selected and reviewed. We show the regulation of oxidative stress, the expression of pro- and anti-inflammatory genes and mediators, and remodeling of the extracellular matrix, expressed by changes in collagen and matrix metalloproteinases levels, in tissues subjected to stretching. We point out that a better understanding of the anti-inflammatory properties of stretching may result in increasing its importance in treatment and recovery from diseases such as osteoarthritis, systemic sclerosis, and cancer.

## 1. Introduction

### 1.1. Inflammation

Inflammation is a universal defensive response of the body to potentially harmful stimuli, where the stimulated macrophages, dendritic cells, and mast cells, through the activation of intracellular signaling pathways, produce eicosanoids and cytokines, mediators of inflammation [1]. Eicosanoids—prostaglandins (PGs), prostacyclins (PGIs), thromboxanes (TXs), leukotrienes (LTs), and lipoxins (LXs)—are produced by the activity of cyclooxygenase 1 (COX-1) (PGs, PGIs, TXs), cyclooxygenase 2 (COX-2) (PGs, LXs), arachidonate 5-lipoxygenase (LTs, LXs), and 12- and 15-lipoxygenase (LXs) from essential fatty acids [1,2]. COX-1 is responsible for maintaining tissue homeostasis through intercellular interactions and for regulating angiogenesis; its expression is constitutive and always at a low level [3]. In contrast, the expression COX-2 is mostly inducible, with this isoform indicated as the major factor in the development of inflammation [4]. Although COX-1 is also involved in inflammation [5], the increase in COX-2 expression and activity is modulated by inflammatory signals and is a more important source of prostaglandins in chronic inflammation than COX-1 [6,7].

PGs synthesized by cyclooxygenases are often called local hormones, and are involved in the control of vasomotor processes and affect autonomic and neuromuscular junctions. They act chemotactically against leukocytes and platelets [8]. In a self-regulating process, prostaglandins secreted by macrophages as a result of tissue injury participate in the formation of inflammation and also lead to the inhibition of the synthesis of macrophage factors such as interleukin-1 (IL-1) and interferons (IFNs), and to the proliferation of macrophage progenitor cells, macrophage migration, and adherence [6,9].

Cytokines—such as interleukins (ILs), colony-stimulating factors (CSFs) and numerous tumor necrosis factors (TNFs)—also regulate inflammatory processes [1]. Anti-inflammatory cytokines IL-4, IL-10, and IL-13 are able to suppress the genes of proinflammatory cytokines such as IL-1, tumor necrosis factor alpha (TNF-α), and chemokines, i.e., substances that can induce the upregulation of proinflammatory genes of phospholipase A2 (PLA2) and COX-2, as well as inducible nitric oxide synthases (NOSs) [10]. Increased expression of these genes enhances the synthesis of platelet-activating factors, leukotrienes, prostanoids, and nitric oxide (NO) [10]. A particular role in the induction of these genes and the initiation of the cascade of inflammatory mediators is played by the proinflammatory cytokines IL-1 and TNF [10].

The interaction of the aforementioned inflammation mediators with their receptors, including IL-1 receptor (IL-1R), IL-6 receptor (IL-6R), and the TNF receptor (TNFR) [11], activates intracellular signaling pathways such as the nuclear factor kappa-B (NF-κB), mitogen-activated protein kinase (MAPK), activator of transcription (STAT), and Janus kinase (JAK)-signal transducer pathways, that regulate inflammatory response by the synthesis of cytokines, the recruitment of inflammatory cells, and/or the regulation of cell proliferation, differentiation, and apoptosis [12].

Inflammation also plays an important role in many stages of tumorigenesis [13,14,15] where it interacts with cellular transformation, promotion, proliferation, invasion, angiogenesis, and metastasis [16]. In the tumor microenvironment formed by cancer, stromal cells, and inflammatory cells, the induction of inflammation follows a distinct timing, and the contribution of tumor-promoting inflammation might emerge prior to or after the initiation of tumorigenesis, and remain silent until the late stages of metastasis [17]. Chronic inflammation may contribute to tumorigenesis [18,19] while, on the other hand, the inflammation is also needed to suppress the growth of the tumor and immunosuppression provides ideal conditions for the growth of abnormal cells, allowing the progression of tumorigenesis [15].

Both pro- and anti-inflammatory factors influence cell activity and function. They constitute a specific regulatory network and condition the proper course and result of inflammatory responses which, if uncontrolled, could lead to irreversible tissue destruction and loss of function [20].

### 1.2. Stretching

Stretching has a positive effect on muscle strength and flexibility, range of motion in a joint, and blood supply to the musculoskeletal system, protecting against injury. In addition, stretching relieves pain caused by excessive muscle tension and is an alternative form of aerobic exercise in diabetics, stroke patients, and oncology patients [21,22,23,24,25,26,27,28,29]. The basis of stretching exercises is brief isometric work of the muscle followed by slow relaxation and stretching for several tens of seconds, which results in an increase in microvascular volume, the number of capillaries per muscle fiber, levels of hypoxia-inducible factor 1 (HIF-1), vascular endothelial growth factor (VEGF), and NOS [28,29,30,31].

Stretching is part of most sports, daily life, and rehabilitation therapies. One of the most widely known and practiced holistic postural traditions is yoga [30], comprising a collection of stretching exercises that involve maintaining postural poses in conjunction with a conscious natural breathing rhythm [31,32]. Tai Chi Chih (TCC), a traditional Chinese art of movement, is also based on stretching, coordination, and relaxation exercises. Exercises such as yoga and TCC lower stress levels, enhance the immune response, reduce circulating IL-6, and increase anti-inflammatory IL-10 levels [33,34,35,36,37,38]. At moderate intensity, they can be successfully performed by older people to improve their health and compensate for a lack of exercise [39].

Stretching is also a regular part of athletic training. Although static stretching during a warm-up can cause performance deficits during exercise [40], it can promote muscle fiber growth which, when combined with strength training, generates an added effect to strength after training. Research suggests that active as well as passive muscle contractions accelerate anabolic pathways in muscles, including the mechanistic rapamycin (mTOR) pathway, which induces protein synthesis and leads to skeletal muscle growth through the activity of phospholipase D (PLD) and phosphatidic acid (PA) production [41,42,43,44]. Physiotherapy and rehabilitation also extensively use techniques based on massage and passive stretching of individual muscle groups [45,46].

Both active and passive stretching have been successfully applied in studies using laboratory animals [47,48,49] and the use of methods involving tensile apparatus allow the mechanical stimulation of selected cell lines such as fibroblasts and chondrocytes [50,51]. Techniques used in the research include myofascial release (MFR), osteopathic manipulative techniques (OMT), similar indirect osteopathic manipulative techniques (IOMT), cyclic tensile strain (CTS), repetitive motion strain (RMS), cyclic short-duration strain (CSDS), and CSDS combined with acyclic long-duration strain (ALDS), which is a basis of MFR [52]. The aforementioned techniques may aim to induce a therapeutic effect (MFR, OMT, CTS) or may cause injury to the tissues under investigation (RMS, CSDS) and subsequent recovery (e.g., ALDS) [52].

The most common stretching method is MFR, a technique that involves applying gentle continuous pressure to myofascial connective tissue, often used in massage-based therapies [53] and OMT, which is a set of practical techniques (articulatory, counterstrain, cranial, facilitated positional release, fascial ligamentous release, functional, low velocity and moderate to high amplitude, lymphatic, muscle energy, myofascial/integrated neuromuscular release, and soft tissue) involving the patient’s muscles and joints undergoing stretching, gentle pressure, and resistance. The precise counterforce applied by a physician allows the specific directed movement of the tissue from a controlled/neutral position in the desired direction [54,55]. Such techniques are often used by osteopathic practitioners and physiotherapists to solve pain and tissue dysfunction and to reduce joint restriction [56]. Indirect osteopathic manipulative techniques (IOMT), similar to MFR, involve holding a musculoskeletal structure in a comfortable position, balancing it in three planes of motion, and continuing to make small adjustments to the position until the tissues around that structure relax [57,58]. A therapeutic effect is also observed after applying cyclic tensile strain (CTS), which simulates continuous passive motion [51].

These therapies have positive results in treating immobility and eliminating skeletal muscle pain by relaxing contracted fibers, improving blood and lymph circulation and oxygen distribution (MFR) [53,58]. They also improve body alignment and mobility (IOMT) and prevent injuries (IOMT) [54,55]. Stretching-based therapies reduce pain levels and improve gait in animal models [47,49,59]. They also lead to pain relief in systemic scleroderma, shoulder, back, and knee pain [46,47,60,61,62,63,64], and also have positive effects in treating insomnia, fatigue, and depressive symptoms [65]. The combination of MFR therapy with conventional medical treatment produces much better clinical results (immediate relief of pain and reduced tissue tenderness) than those that occur with drug therapy alone [66,67]. In addition, the use of stretching techniques leads to reduced swelling, reduced analgesic dosage, and increased range of motion, as well as improved joint and muscle conditioning after injury [68,69,70].

It is well-documented that correctly guided rehabilitation helps to minimize or slow down the disabling effects of chronic health conditions [60,71,72]. Most likely, these benefits are due to minimizing inflammation in response to stretching exercises. Recent studies demonstrate the positive effects of stretching not only on systemic but also localized inflammation—resulting in reduced inflammatory infiltration around subcutaneous lesions, neutrophil count and migration, and inflammatory lesion thickness [47,49]. However, the molecular and tissue mechanisms underlying the anti-inflammatory properties of stretching-based exercise are still poorly understood. Such understanding may contribute to the significance of stretching and its addition to the pharmacological treatment of conditions caused by generalized and local inflammation.

To date, there has not been a review summarizing current knowledge on the mechanisms underlying anti-inflammatory and anti-cancer properties of stretching. Therefore, the aim of this review is to collect and summarize available literature in the area in terms of morphological changes, regulation of proinflammatory genes and cytokine expression in inflammation induced by applying RMS and CSDS, the introduction of IL-1 or carrageenan, as well as collagen synthesis and degradation and its anti-cancer properties.

A comprehensive search of PubMed, ScienceDirect, and Google Scholar was conducted from 2000 to 2022 to identify suitable literature. The search strategy included the following terms: ((stretch) OR stretching) AND anti-inflammatory AND (in vivo or in vitro or ex vivo or fibroblasts or chondrocytes). From 1081 identified studies, only 22 met the criteria. Inclusions required stretching to be applied as a treatment method, the existence of a control group, and evaluation of at least one inflammation-related parameter (i.e., macroscopic/microscopic evaluation, inflammatory cell count/sorting, enzymatic techniques, gene/protein expression evaluation, etc.). Publications that did not refer to the anti-inflammatory effect of stretching, as well as studies that included stretching of other body systems (i.e., vascular system, respiratory system, etc.), were excluded. The results were organized into sections, which enabled assessment of the effects of stretching at the macroscopic and microscopic levels (inflammatory lesion and tissue morphology) and at the molecular level (proinflammatory genes and cytokine expression), and divided into results obtained on patients, animals, and cell cultures. Then, we reviewed the effect of stretching on collagen formation and degradation, along with its effect on systemic sclerosis (SS) and the anti-cancer effect of low-level exercises.

## 2. Stretching and Inflammation

### 2.1. Inflammatory Lesion and Tissue Morphology

The inflammatory infiltrate formed by the infiltration or accumulation of inflammatory cells is the result of tissue reactions to harmful stimuli. The infiltrate is formed by the action of neutrophils, lymphocytes, plasmacytes, eosinophils, macrophages, and mast cells and is accompanied by the vasodilation and accumulation of exudate, resulting in tissue swelling [73].

Corey et al. (2012) showed that stretching reduces inflammatory infiltration around subcutaneous lesions, improves gait, and reduces pain sensitivity. In their model, inflammation was induced by injecting carrageenan (a polysaccharide that, when injected subcutaneously, causes severe swelling) into the subcutaneous connective tissues of the lower back of studied animals (mice). The stretching model used was similar to methods used in physiotherapy or yoga (active stretching) [47], and the effect of the exercises performed was a significant change in macrophage marker expression (CD68) around the tissue analyzed. The carrageenan/stretch group had reduced macrophage marker expression in the connective tissue of the lumbar region compared to the carrageenan/no-treatment group and the carrageenan/sham group. Ultrasound measurements additionally showed a reduced thickness of the examined tissues, understood as the size of the inflammatory infiltrate in the stretch group [47].

In another study using the carrageenan inflammation model, Berrueta et al. (2016) demonstrated that active and passive stretching (under anesthesia) activates local anti-inflammatory processes in mice [49]. Ultrasound results showed that stretching reduced the thickness of the inflammatory lesion and its cross-sectional areas, as well as reduced the number of neutrophil granulocytes and the total number of cells in the inflamed area. Both passive and active stretching produced similar results, and these effects were similar to those observed after treatment with resolvin D2 (Rvd2), a specialized pro-resolving mediator (SPM) with anti-inflammatory properties [49,74]. SPMs are a wide group of cell signaling agents produced from polyunsaturated fatty acids (PUFA). They take part in the resolution and silencing of the inflammatory response by inhibiting the migration and/or infiltration of inflammatory cells and the release of proinflammatory mediators [75,76]. Researchers also conducted ex vivo studies to investigate the local effects of tissue stretching independent of the vascular, lymphatic, and neuromuscular systems. They showed that tissue stretching 48 h after carrageenan injection was associated with a significant reduction in neutrophil migration in the connective tissue in mice [49]. Similar results were obtained by Wang et al. (2022) where, in a posttraumatic knee contracture model, the implementation of static progressive stretching (30 min) resulted in a reduction in the number of inflammatory cells [59].

Looking for the basis of the analgesic action of MFR, Meltzer et al. used the RMS-induced inflammation model in human fibroblast cultures [50]. They observed elongated lamellopodia, cellular decentralization, larger intercellular distances, and reduced cell–cell contact area, which resulted in induced inflammatory responses. Reductions in the fibroblast structure were noticed following MFR therapy (3 h after RMS). The apoptosis rate in the RMS group was elevated, and the implementation of MFR reduced this. The application of both techniques did not alter the proliferation rate of fibroblasts [50].

In 2018, Langevin et al. used domestic swine to study the effect of stretching on inflammation, as their back structure is more similar to humans than were the rodents used in previous studies [47,49]. The animals of the study groups underwent a unilateral fascia injury in the dorsal trunk, then were subjected to movement restriction (prevented from full hip extension and pelvic lateral flexion in the transverse plane during gait) and then stretching. The stretching model differed from those used in previous studies [47,49] because the focus was on stretching the hip and lower back [77]. The fascia thickness increased overall from week 8 to week 12, despite the animals returning to normal gait speed. However, this effect may be linked to non-inflammatory pathologies and physiological processes such as tissue growth, muscle contraction, and mucosal physiology [78]. By week 12, there were no significant differences in fascia thickness between the groups. This shows that reduced fascia mobility in response to an injury along with movement restriction is a plastic phenomenon that worsens over time and persists even when movement is restored. Four weeks of daily 10 min passive stretching of the hip and lower back tissues after the restriction was removed was not superior to simply removing the restriction [77]. Despite the lack of difference between the stretching and non-stretched groups, the study suggests that an important issue in controlling inflammation through stretching may be the stretching method itself, with the method used not exerting sufficient tension on the thoracolumbar fascia [77].

Studies using domestic swine were also conducted by Vergara et al. (2020), this time using stretching techniques similar to those used by Berrueta et al. and Corey et al. [47,49]. A carrageenan-induced inflammation model was used and the pigs were stretched by holding their legs twice a day for 5 min over 48 h [48]. The results obtained showed reduced inflammatory lesions and lesion mass in the stretching (S) group compared to the non-stretched (NS) group. The S group’s lesions had 71% fewer granulocytes and 49% fewer macrophages compared to the NS group. However, the observed differences were not statistically significant (probably due to the small size of the study groups, *n* = 4 for each group) [48].

The results obtained in the aforementioned studies confirm that stretching affects local inflammation and accelerates the resolution of inflammation [47,49]. They also indicate a significant role of rehabilitation and stretching in reducing inflammatory infiltration, which is likely underpinned by both systemic and local mechanisms [47,49]. Furthermore, the mode of stretching seems to be significant [48,77]. Stimulation of connective tissue may be an important therapeutic goal, and stretching may serve as a viable method of treatment [49,50].

### 2.2. Proinflammatory Genes and Cytokine Expression

Most proinflammatory genes are not expressed under physiological conditions but are rather controlled by phosphorylation and dephosphorylation of many transcription factors, and can be triggered by stress factors that activate intracellular signaling pathways such as mitogen-activated protein kinase (MAPK) cascades, the NF-κB, and the JAK–STAT signaling pathway [10,12,79]. Dysregulation of these genes is associated with inflammation and the progression of diseases such as cancer, diabetes, and autoimmune diseases [79,80]. The cytokines secreted as a result of inflammatory response also activate the aforementioned cascades [10].

#### 2.2.1. Patient Studies

Stretching exercises, as well as exercises with a prominent stretching component (e.g., yoga, Tai Chi), can reduce levels of circulating proinflammatory cytokines [35,65,81,82]. A study by Sarvottam et al. (2013), based on hourly whole-body stretching exercises (yoga) performed for 10 days, showed a reduction in IL-6 levels as well as increased levels of adiponectin—a potential endogenous anti-atherogenic factor produced by mature fat cells [81,83]. Overweight and obese patients often exhibit low-grade inflammation, which can result in chronic inflammatory disease [84]. These patients also often have elevated circulating IL-6, a proinflammatory interleukin that is a risk indicator for cardiovascular disease (CVD) [85]. According to Sarvottam et al. (2013), even a short-term change in lifestyle can lead to lower blood pressure and weight loss, and can exert anti-inflammatory and anti-atherogenic effects. Despite the study being conducted on a group of only 51 men, the results obtained indicate the relevance of stretching exercises in reducing the risk of CVD [81]. Similar results have been obtained by trainers of TCC, which is shown to be a useful behavioral intervention resulting in lower circulating levels of IL-6 [35].

#### 2.2.2. Animal Studies

The study mentioned earlier in Section 2.1 was based on a carrageenan-induced inflammation model [49] in which stretching resulted not only in morphological improvements, but also in higher concentrations of resolvin D1 (RvD1), a signaling molecule with anti-inflammatory properties [86,87,88]. The concentration of LTB4, an eicosanoid produced during inflammation [89], was not significantly altered by stretching. However, the studies showed a two times higher ratio of RvD1 to LTB4 following stretching, compared to controls, thus demonstrating the additional anti-inflammatory potential of stretching [49].

Vergara et al. conducted a study on pigs (*n* = 4) to analyze the production of SPMs in carrageenan-induced inflammation and found no changes in the expression of any of the investigated proinflammatory genes, with differences in protein expression not statistically significant, most likely due to the small group size [48]. However, some trends were observed. In the serum, both lipoxin A4 and RvD1 were higher in the stretching group, with the proinflammatory mediator prostaglandin D2 (PGD2) exhibiting an almost twofold decrease. Within the lesion, the stretching did not alter RvD1 or LXA4 levels; however, the ratio of serum LXA4 or RvD1 to PGD2 showed a nearly twofold increase following stretching compared with the control group [48]. Lipoxins are specialized pro-resolving mediators (SPMs), and their synthesis increases in response to increased concentrations of arachidonic acid metabolites [74]. The increased ratio between LXA4 and PGD2 could, therefore, suggest the initiation of resolution of the inflammation.

#### 2.2.3. Cell Culture Studies

##### Mechanically Induced Inflammation

Meltzer and Standley (2007) used RMS in their study to model IOMT and investigate the response of fibroblasts [90]. OMT is a set of techniques used by osteopathic physicians, during which elements of stretching are combined with the application of appropriate pressure to muscles and joints [91], and is often prescribed for the management of many health conditions [92]. RMS in the fibroblast culture resulted in decreased cell proliferation, decreased secretion of interleukin-1 receptor antagonist (IL-1ra) with anti-inflammatory properties, and increased secretion of proinflammatory factors IL-1α, IL-1β, IL-2, IL-3, IL-6, and IL-16. In contrast, IOMT treatment alone resulted in a decrease only in the secretion of the proinflammatory IL-3 [90]. IOMT following stimulation of the RMS cells did not lead to an induction of interleukin secretion that could be observed after RMS alone, but resulted in a decrease in proinflammatory IL-6 secretion and an increase in cell proliferation. Thus, the inflammatory response of fibroblasts is dependent on the stretching model used, with IOMT appearing to reverse the proinflammatory effects caused by RMS stimulation by regulating cytokine secretion [90].

Eagan et al. (2007) investigated the cellular mechanisms behind the positive clinical outcomes of manual medicine treatments (MMT) and showed that regulation of the inflammatory response was influenced by the stretching model used, with equibiaxially strained cells having increased fractalkine (CX3CL1) secretion [93]. A soluble fraction of fractalkine serves as a chemoattractant for T cells, monocytes, and NK cells [89]. The aforementioned cytokine regulates apoptosis and can promote the death of damaged neural cells [94], but when released from apoptotic cells, it induces both antiapoptotic and mitogenic effects on neighboring vascular smooth muscle cells, and promotes proper wound healing and regeneration by inhibiting fibrotic responses to cell death [95,96]. MMT was also associated with reduced secretion of the pulmonary and activation-regulated chemokine/CCL18, as well as the macrophage-derived chemoattractant/chemokine MDC and, compared to the model of heterobiaxial strain, equibiaxially stretched cells showed reduced proliferation and reduced secretions of MDC and IL-6 [93].

Meltzer et al. (2010) observed an increased apoptosis rate in RMS-induced inflammation, which was most likely based on the observed upregulation of apoptosis-associated signaling protein kinase 2 (DAPK-2). After implementing MFR as a treatment for inflammation, a downregulation of this protein was observed, as well as an increase in serine 133-phosphorylated cyclic adenosine monophosphate (cAMP) response element-binding protein (CREBS133) in the RMS groups [50]. Phosphorylation of CREB at site S133 activates this protein, leading to an altered expression of many genes [97]. It has been proven that overexpression of CREB protects against tunicamycin-induced apoptosis [98]. Upregulation of phosphorylated CREB also increased in the MFR and RMS+MFR vs. RMS groups, which may indicate the implementation of processes aimed at limiting apoptosis, the rate of which is faster following MFR. A similar effect may arise from the upregulation of phosphorylated focal adhesion kinase (FAK), an enzyme suppressing apoptosis [99], observed both in RMS vs. control and following MFR in groups with no inflammation and the RMS-induced inflammation group [50]. The lack of change in the proliferation rate in this study was probably masked by DAPK-2-associated apoptosis [50]. In terms of the secretion of cytokines and growth-promoting mediators, Meltzer et al. (2010) observed no changes indicative of modulation of the inflammation but, as they noted, their results do not exclude the existence of changes in the expression of receptors of the studied mediators, as well as in the expression of intracellular effectors or expression/secretion in non-measured mediators [50].

Another study was undertaken by Anloague et al. 2020, analyzing the effect of mechanical stimulation of human dermal fibroblasts on inflammatory processes, wherein primary human dermal fibroblasts were subjected to an 8-hour-long CSDS or CSDS combined with ALDS. Anloague et al. confirmed that cyclical mechanical strain increases levels of IL-6 and, adding long-duration stretching intended to mimic therapeutic soft-tissue stimulation, results in a reduced IL-6 levels [100]. Expanding the cytokine profile also allowed the team to prove that long-duration stretching (CLDS+ALDS) results in lowered levels of IL-8, one of the most potent chemotactic factors [100]. Similar results were obtained by Nazet et al. (2020), where advanced stretching led to a reduction in the inflammatory effects of TNF-α, IL-6, and IL-1β, but the implementation of short-term high-frequency cyclic tension or static isotropic tension was associated with proinflammatory effects [101].

##### IL-1-Induced Inflammation

More than any other cytokine family, the IL-1 family is primarily associated with innate immunity [102] and IL-1β is often used to induce inflammation in studies on rodents [51,103,104,105,106].

In a study on rabbit articular cartilage, Xu et al. (2000) demonstrated that CTS acts as a potent antagonist of IL-1β [51]. High levels of IL-1 in the joint synovium of patients with osteoarthritis are associated with cartilage destruction [107,108]. IL-1 increases leucocyte recruitment and increases the activity of matrix metalloproteinases (MMPs), leading to joint destruction through both degradation and decreased synthesis of matrix components [109]. Due to the increased production of reactive nitrogen species, IL-1 is also associated with elevated deoxyribonucleic acid (DNA) damage [110]. In contrast, stimulation (CTS) of chondrocytes cultured in the presence of IL-1β leads to the suppression of the expression of proinflammatory genes such as inducible nitric oxide synthase (iNOS) and COX-2 and, consequently, to a decrease in the synthesis of NO and prostaglandin E2 (PGE2). The anti-inflammatory and regenerative effects observed following CTS are similar to those obtained with drugs that reduce cartilage degradation [51].

Further studies on the mechanisms responsible for the anti-inflammatory properties of CTS were conducted by Madhavan et al. in 2006. They cultured CTS chondrocytes in the presence of IL-1β for various periods, followed by a period of rest. The researchers showed that 90% of the expression of IL-1β-induced proinflammatory genes—iNOS, COX-2, matrix metalloproteinases 9 (MMP-9), and matrix metalloproteinases 13 (MMP-13)—was able to be blocked by continuous CTS [111]. Eight hours of CTS was able to reverse the changes produced by 16 h of exposure to IL-1β, but was unable to reduce iNOS expression after 28 h and 40 h of exposure. The data suggest that continuous CTS inhibits IL-1β-induced proinflammatory gene expression at the transcriptional level, and that the signals generated by CTS are sustained after cessation, with the persistence depending on the duration of exposure [111].

**Figure 1:**
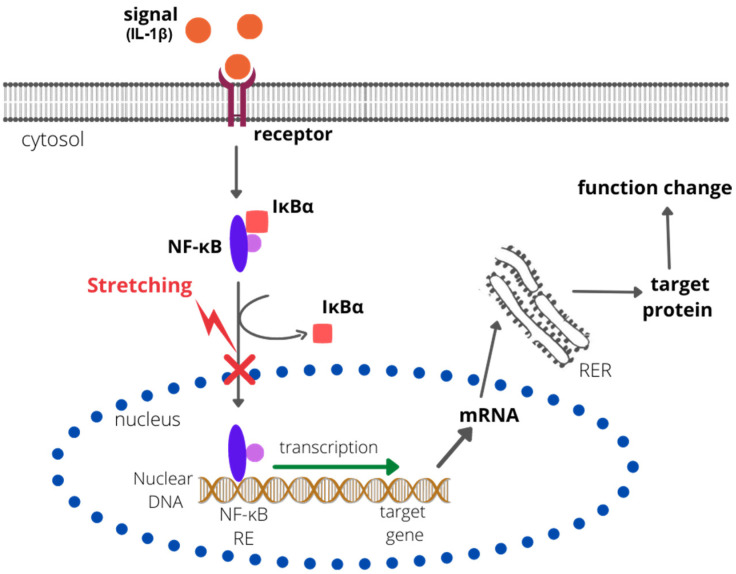
The effect of CTS on the NF-κB pathway. CTS inhibits the degradation of IκBα and IκBβ and rapidly inhibits the IL-1β-induced nuclear translocation of NF-κB. Therefore, the NF-κB response element (NF-κB RE) is not bound by the NF-κB complex, the transcription is not activated, and no target protein is synthesized. RER—rough endoplastic reticulum; IκBα/β—nuclear factor of kappa light polypeptide gene enhancer in B-cell inhibitors alpha/beta).

Research on the effects of biomechanical signals in joint inflammation was also conducted by Dossumbekova et al. (2007), who, using a rat chondrocyte culture, demonstrated that CTS inhibits the IL-1β-induced activation of proinflammatory genes by the nuclear factor kappa-light-chain-enhancer of activated B-cell (NF-κB) cascades [112]. The analysis by Dossumbekova et al. (2007) shows that CTS rapidly inhibited the IL-1β-induced nuclear translocation of NF-κB, but not its phosphorylation at serine 536 and serine 276. Scientists also showed that the stretching method they used repressed gene transcription of IκBα and IκBβ (associated with the NF-κB pathway); however, it inhibited their cytoplasmic protein degradation. The reduction in degradation of IκB was caused by downregulation of IκB kinase activity. A rapid nuclear translocation of IκBα, presumably to prevent the binding of NF-κB to DNA, was also observed [112]. The results indicate that the NF-κB signaling cascade is indirectly affected by CTS at multiple points (sites), resulting in the attenuation of IL-1β-induced proinflammatory gene expression [112] (Figure 1).

Similar results were obtained by Branski et al., 2007, using rabbit vocal cord fibroblasts cultured in the presence of IL-1β following different magnitudes of CTS [113]. The results obtained confirm earlier reports on the increase in expression of iNOS, COX-2, and MMP-1 in vocal cord fibroblasts at the mRNA and protein level under the influence of IL-1β. CTS nullified the IL-1β-induced activation of the mentioned genes in a magnitude-dependent manner [113].

Mechanical signals of low magnitudes applied in dynamic mechanical stimulation show a strong anti-inflammatory potential. Their application leads to the attenuation of proinflammatory gene induction by IL-1β and TNF-α [114,115]. A study on the time-dependent effects of dynamic tensile forces (DTF) on fibrochondrocytes harvested from rat knees by Ferretti et al. (2006) showed, similarly to Madhavan et al. (2006), inhibition of IL-1β-dependent induction of iNOS [115]. The observed effect depended on the magnitude used and was present for up to 20 h after the end of stimulation. The mRNA expression of IL-1β decreased successively after the application at magnitudes ranging from 5% to 20%, which translated into NO accumulation as well as iNOS synthesis in IL-1β-induced inflammation. The results obtained by Ferretti et al. were also dependent on the frequency of the signals used—the greatest decreases in iNOS mRNA expression were observed at the lowest frequency applied—0.025 Hz. At other applied frequencies, the decrease in iNOS expression was not as spectacular. DTF also strongly inhibited the mRNA expression of TNF-α and MMP-13 and their proteins. An increase in the expression of these molecules is observed after injury and, therefore, the introduction of DTF blocks the expression of inflammatory mediators and protects inflamed joints from a loss of function. The results reported by Ferretti et al. in 2006 indicate that mechanical signals act as strong anti-inflammatory signals and this response is magnitude- and frequency-dependent and continues even after DTF cessation. The use of mechanical forces of appropriate intensity may, therefore, be recommended in the rehabilitation of meniscal cartilage [115].

The aforementioned studies confirm that CTS and DTF reduce cartilage degradation, producing effects similar to those observed following pharmacotherapy. IL-1β-induced inflammation under stretching may be reduced by changes in the expression of iNOS and COX-2, MMP-9 and MMP-13, MMP-1, and TNF-α, causing a decrease in NO and PGE2 synthesis, and affecting the NF-κB signaling cascade [111,112,113,115]. The presence of IL-1β in the joint synovium of patients with RA or osteoarthritis (OA) plays a key role in cartilage destruction [103,107,108]. Therefore, CTS may be particularly important in alleviating and controlling arthritic diseases of different etiologies. Further research is required to more precisely establish the molecular consequences of tissue stimulation by stretching.

## 3. Stretching and Collagen Metabolism

### 3.1. Collagen Synthesis and Degradation

Collagen is the major insoluble fibrous protein present in the extracellular matrix and connective tissue, with aggrecan being the major proteoglycan in articular cartilage [116]. During inflammation, collagens are able to modulate the cellular inflammatory response and activity, according to the microenvironment and physiological processes involved [117]. MMPs, collagen-degrading enzymes, are involved in this regulation. Aggrecan interacts with the morphogens and growth factors directing tissue morphogenesis, remodeling, and metaplasia [118]. Mediators associated with inflammation and joint strain degrade the aggrecan, with the presence of aggrecan fragments as a marker of ongoing cartilage destruction in osteoarthritis. Aggrecan participates in both the demise and survival of articular cartilage [119,120]. Collagen type II and aggrecan are important structural components of cartilage, but the increased accumulation of collagen in the skin and other tissues can lead to impaired tissue function and cause diseases such as systemic sclerosis (SS) [71,120].

A study on articular cartilage [51] showed increased collagen production in response to CTS, the suppression of IL-1β-dependent collagenase synthesis, and a reversal of IL-1β-induced downregulation of tissue inhibitor of metalloproteinases 2 (TIMP2). Under these conditions, enzymatic degradation of collagen type II is reduced and new collagen molecules can improve the condition of the joint [51]. Furthermore, the synergistic action of CTS and gallic acid resulted in the increased deposition of glycosaminoglycan and collagen type II and IX in human articular chondrocytes [121].

A study by Bouffard et al. (2008) was based on the premise that transforming growth factor β1 (TGF-β1) is one of the most important cytokines regulating the fibroblast response to injury, affecting remodeling, immune modulation, scarring, fibrosis, and development or progression of cancer [122,123,124,125]. Bouffard et al. (2008) tested if a 10-minute-long static tissue stretch attenuated TGF-β1-induced procollagen formation. They used an ex vivo model (mouse subcutaneous tissue) and an in vivo model—mice with a unilateral subcutaneous microsurgical back injury, where mice were subjected to stretching for 4 or 7 days, 10 min per day, respectively. In the ex vivo study, TGF-β1 protein levels were lower in the stretched tissue compared to the unstretched tissue, whereas in the in vivo model, the microinjury caused a significant increase in type-1 procollagen levels in the unstretched group but not in the stretched group [122]. It is well documented that long-term low amplitude static or cyclical stretching in a TGFβ-induced inflammation model increases the synthesis and deposition of collagen [126,127,128,129,130]. In contrast, Bouffard et al. (2008) demonstrated that short-term tissue stretching attenuates an increase in both soluble TGF-β1 (ex vivo) and type-1 procollagen in connective tissue after injury [122]. The results are consistent with those obtained by Wang et al. (2022), where applying static progressive stretching in rats with traumatic knee contracture lowered the expression of TGF-β1 and IL-6, and suppressed collagen proliferation [59,131]. Since the increase in expression of TGFβ mRNA, similar to type-1 procollagen, occurs following an injury [132], the results obtained by Bouffard et al. and Wang et al. indicate that inflammation is attenuated after the application of a brief static tissue stretch [122,131].

In vocal fold fibroblasts, in which type-I and type-IX collagen are the major collagens, Branski et al., 2007 demonstrated that CTS blocks IL-1β-induced inhibition of collagen synthesis, increasing the amount of collagen type I. The authors state that their results present the beneficial effects of low-level vocal exercise and their importance in tissue regeneration [113].

Stretching also modulates the aggrecan concentration. In a study on chondrocytes [111], 3% CTS at 0.25 Hz was shown to negate the IL-1β-induced inhibition of aggrecan synthesis. In addition, even after a 20-hour-long rest following CTS, a decrease in the expression of MMP-9 and MMP-13 was observed, indicating the inhibition of collagen degradation. In addition to collagen, MMP-13 activity also leads to the degradation of aggrecan, so a decrease in the expression of this metalloproteinase will be an additional factor in protecting the tissue from damage [133]. The remodeling of the extracellular matrix (decrease in collagen-I-alpha-2) was also observed by Nazet et al. after applying long-term tensile strain [101], and by Abusharkh et al. (2021), where combination of gallic acid and CTS led to the downregulation of MMP-1 and MMP-13 [121]. The findings prove that the effectiveness of physiotherapy at the cellular level may be relevant in the management of arthritic joints [111].

Increased collagen synthesis and MMP expression are observed following injury and are associated with ongoing inflammation. During tissue repair and scar formation, however, excess deposition of fibrous connective tissue can lead to impaired tissue function. The anti-fibrotic or pro-fibrotic effect of stretching is probably dependent on the amount, timing, and duration of the therapeutically applied stretch.

### 3.2. Systemic Sclerosis

Systemic sclerosis (SSc) is an autoimmune disease characterized by increased collagen accumulation in the skin and other tissues [134]. It targets the vascular system, immune system, and connective tissue fibroblasts and myofibroblasts. Treatment of this incompletely understood disease remains a challenge for clinicians and new therapeutic approaches are constantly being sought [134,135].

In a 2017 study, based on previous findings that stretching promotes the resolution of inflammation [47,49] and lowers the formation of collagen after injury [122], Xiong et al. analyzed whether stretching could also delay the development of SSc. To induce dermatitis followed by fibrosis, Xiong et al. used the adoptive transfer of splenocytes from B10.D2 mice into Rag2^−/−^ BALB/c hosts (sclerodermatous graft-versus-host disease (scl-GvHD)) [71]. The use of scl-GvHD allowed researchers to map the inflammation observed in SSc patients [136,137]. After 3 weeks of in vivo experimentation, a decrease in the thickness of the analyzed tissue and greater relative tissue displacement in scl mice undergoing stretching were observed, indicating the extinction of the inflammatory response in the analyzed area. Suggesting a lack of difference in the simple displacements of either skin or subcutaneous tissue, Ying Xiong et al. (2017) inferred that stretching does not change the absolute amount of tissue displacement, but affects the relative inter-layer mobility of tissues [71]. In contrast, an ex vivo experiment showed that 4 weeks of stretching reduces fibroblast expansion in explants from scl-GvHD mice, and in vivo stretching does not prevent the loss of fibroblast responsiveness ex vivo [71]. In earlier reports, scientists showed that ex vivo stretching of fibroblasts causes a decrease in tissue tension and an expansion of the cell cytoskeleton [75]. However, in the Xiong et al. study, impairments in the remodeling of connective tissue fibroblasts observed in scl-GvHD explants were not affected by stretching, which suggests that the beneficial effect of stretching in SSc is not based on fibroblast-mediated tissue relaxation [71].

Xiong et al. (2017) also examined the expression of TGF-β, a tissue inhibitor of metalloproteinases 1 (TIMP1), MMP-12, a disintegrin and metalloproteinase domain-containing protein 8 (ADAM8), interleukin-4 receptor subunit alpha (IL4RA), and chemokine (CC motif) ligand 2 (CCL2)—factors that are upregulated in both mouse scl-GvHD and the inflammatory subgroup of SSc patients [136,137,138,139]. Stretching significantly decreased ADAM8 and CCL2 mRNA expression [71].

The aforementioned studies indicate that daily stretching, even in the absence of drug treatment, may contribute to a reduction in inflammation in a mouse model of scl-GvHD. Stretching may, therefore, be part of therapy in patients with SSc.

## 4. Stretching vs. Cancer

Physical activity is associated with increased survival in many cancer types. Cancer patients practicing yoga, TCC, and Qi Gong have reported improved mobility and well-being [21,23,24]. Stretching exercises following breast cancer surgery resulted in increased shoulder range of motion and decreased chest tightness and pain—these exercises are an important part of rehabilitation to prevent post-surgical complications [140,141]. It is also known that intensive exercise slows tumor growth [142,143], reduces intratumoral inflammation [72,144], and inhibits the progression of androgen-dependent prostate LNCaP tumors [145]. However, heavy physical exercise is often impossible for cancer patients, so stretching may provide a good alternative, although the molecular basis of the beneficial effects of physical activity on attenuating tumor growth is still undefined. To date, only one study considering the molecular actions of stretching in cancer patients has been conducted.

**Figure 2:**
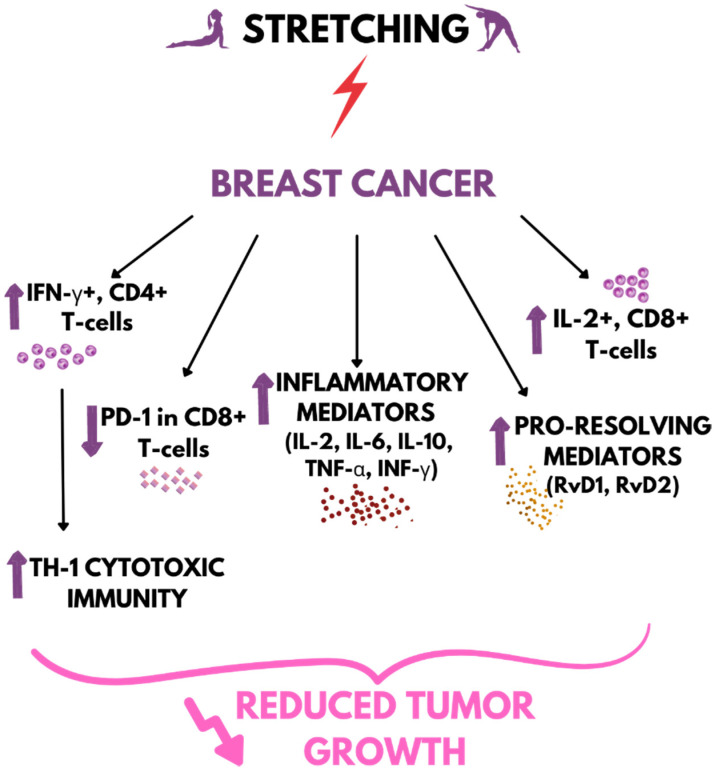
The effect of stretching on inflammatory factors in breast cancer mice. ↑ increase/upregulation; ↓ decrease/downregulation.

In a study by Berrueta et al. (2018), a mouse breast cancer model was obtained by giving mice a bilateral injection of p53/PTEN double-null primary mouse mammary tumor cells. The researchers used an earlier stretching model [49] involving raising the animals by the base of the tail to a ~45° angle to horizontal. The results showed no significant difference in the abundance of macrophages expressing clusters of differentiation (CD) 64 and CD206/Arg between the groups; however, tumor growth from week 2–4 was slower in the stretching mice, and the tumor volume at euthanasia was more than half that of the non-stretching group. Overall levels of inflammatory mediators, including IL-2, IL-6, IL-10, TNF-α, and INF-γ, were elevated in the stretching group (Figure 2) [146].

In a model of carrageenan-induced inflammation, stretching was confirmed to increase the number of INF-γ^+^ CD4^+^ T cells within the inflammation [146]. INF-γ is a cytokine mainly produced by natural killer (NK) cells and natural killer T (NKT) cells, as well as CD4 Th1 and CD8 cytotoxic T lymphocyte effector T cells, and is involved in cytotoxic immune responses [147]. Thus, the increase in INF-γ in the stretching group indicates that stretching promotes TH-1 cytotoxic immunity. During tumor development, cytotoxic immunity is impaired, resulting in T-cell exhaustion. The reduction of cytokines and the presence of inhibitory receptors on T cells reduce the destruction of cancer cells, allowing them to proliferate [148]. A study by Berrueta et al. (2018) showed no differences in total numbers of CD3^+^, CD4^+^, and CD8^+^ tumor-infiltrating T cells, but the expression of programmed death receptor-1 (PD-1) in CD8^+^ T cells was lower in the stretching mice compared to the control mice. The isolates of CD8^+^ T cells from the tumor-draining axillary lymph node showed no differences in TNF-α^+^ and INF-γ^+^ lymphocytes; however, IL-2^+^CD8^+^ lymphocytes were significantly more abundant in the stretching group. The study also described the levels of RvD1 and RvD2, pro-resolving mediators, as significantly greater in the stretching mice vs. the control mice (Figure 2) [146].

Chronic inflammation may contribute to tumorigenesis [18,19]; however, the inflammatory process is also needed to suppress the growth of the tumor, and immunosuppression provides ideal conditions for the growth of abnormal cells, allowing the progression of tumorigenesis [15]. Therefore, the results of Berrueta et al. indicate that stretching restores cytotoxic immunity by reversing CD8^+^ T-cell impairment and T-cell activation. The interaction of cytotoxic immunity and pro-resolution mechanisms involving the action of RvD1 and RvD2 may thus contribute to reduced tumor growth in response to stretching [146].

## 5. Conclusions and Future Prospects

The beneficial effects of stretching are the basis of its use in physiotherapy and rehabilitation. Recent studies show that the activation of both systemic (reducing the level of circulating proinflammatory cytokines) and localized anti-inflammatory mechanisms are responsible for these results. In tissues subjected to stretching, a decrease in inflammatory infiltration is observed, as well as a decrease in the number and migration of neutrophils, which may be caused by changes in the expression of iNOS and COX-2, MMP-9 and MMP-13, MMP-1, and TNF-α, causing a decrease in NO and PGE2 synthesis, as well as the effect of stretching on the NF-κB signaling cascade. The model of stretching seems to be significant—active stretching has significantly greater benefits than passive stretching [146], and CTS and DTF reduce cartilage degradation, producing effects similar to those observed in pharmacotherapy. Further, the anti-fibrotic or pro-fibrotic effect is most likely dependent on the amount, timing, and duration of the therapeutically applied stretching. Revised publications have been summarized in Table 1.

Studies that involved whole-body stretching (patient studies and animal active stretching) have some additional limitations. It is not possible to distinguish between a local and general effect of stretching on localized inflammation. For example, myokines, inflammation regulators released by contracting muscles which balance skeletal muscle metabolism, and take part in the crosstalk between muscles and short- or long-distant organs, are a crucial action during different stages of muscle development [149]. Therefore, during stretching, myokines may also modulate the injury-induced inflammation site localized outside the muscle.

Muscle-derived IL-6, one of the myokines with anti-inflammatory properties [150], inhibits TNF production and stimulates the production of IL-1ra and IL-10 as well as cortisol production, leading to lymphopenia and neutrocytosis. Cortisol itself is a potent anti-inflammatory molecule that prevents tissue and nerve damage. IL-1ra, as mentioned before, is an important inhibitor of IL-1β signal transduction; additionally, IL-10 decreases the synthesis of TNF-α [151], while IL-6 derived from myeloid cells and muscle suppresses macrophage infiltration of adipose tissue [152].

Additionally, stretching increases the microvascular endothelial function by minimalizing capillary diameter, leading to enhanced angiogenesis by increasing HIF-1alpha and VEGF-A expression. The hyperemia after a stretch causes an increased influx of Ca^2+^ and the production of NO, a potent vasodilator. Improving the circulation around the inflamed tissue may also contribute to the observed anti-inflammatory properties of stretching.

Exercise training leads to a decrease in adipose tissue [153] and, hence, a decrease in circulating inflammatory factors, which may also modulate inflammatory processes. Additionally, exercising leads to hormone release, which may also affect ongoing inflammation. Yoga stretching contributes to lowering cortisol levels and increasing testosterone levels in saliva [34]. Despite cortisol’s anti-inflammatory properties, prolonged cortisol exposure may lead to compensatory downregulation or resistance of the glucocorticoid receptor and its binding to the mineralocorticoid receptor, associated with the proinflammatory response [154]. Furthermore, increased levels of testosterone have been linked with lowered levels of inflammatory cytokines [155]. Therefore, lowering the basic cortisol level and increasing testosterone levels may contribute to a better anti-inflammatory response during the injury. Six months of yoga practice may also affect thyroid metabolism, which can also modulate the inflammatory response of the organism. Other hormones such as epinephrine or estradiol, which are beneficial to muscle strength [156], might also take part in that modulation; however, these changes tend to be present in long-term exposure [157,158].

Studies have not been conducted on the influence of stretching on myokines and hormone release associated with their anti-/proinflammatory actions in connective tissue. In a living organism, a whole-body response to inflammation is inevitable; however, in vitro studies do show the beneficial effect of stretching in localized inflammation.

The aforementioned studies suggest that connective tissue stimulation may be an important therapeutic goal, stretching may serve as a method of treatment in the alleviation and control of arthritic diseases of various etiologies, and daily stretching, even in the absence of pharmacological treatment, may contribute to a reduction in inflammation in SSc. Stretching may also restore cytotoxic immunity, which—acting with pro-resolution mechanisms involving the action of RvD1 and RvD—may, therefore, contribute to a reduction in tumor growth.

## Figures and Tables

**Table 1:** Table summarizing anti-inflammatory effects of stretching.

Treatment/Model	Results	References
Inflammatory Lesion and Tissue Morphology		
Active and passive stretching/carrageenan-induced inflammation	↓ CD68 expression (macrophages number) ↓ thickness of the inflammatory lesion ↓ lesion mass ↓ number of neutrophil granulocytes and the total number of cells in the inflamed area ↓ neutrophil migration	[47,48,49]
Static progressive stretching/post-traumatic knee contracture model	↓ number of inflammatory cells	[59]
MFR/RMS-induced inflammation, fibroblasts	↓ intercellular distances	[50]
Passive stretching/unilateral fascia injury	↑ fascia thickness from week 8 to 12	[77]
Static tissue stretch/dermatitis followed by fibrosis (systemic sclerosis-like inflammation)	↓ thickness of the tissue greater relative tissue displacement ↓ fibroblast expansion ex vivo	[71]
**Proinflammatory Genes and Cytokine Expression and Collagen Metabolism**		
***Patient studies***		
Yoga-based exercises, TCC/human studies	↓ IL-6 levels in serum ↑ levels of adiponectin in serum	[35,81]
***Animal studies***		
Active stretching/carrageenan-induced inflammation,	↑ lipoxin A4 and RvD1 [48] ↓ prostaglandin D2 (PGD2) ↑ the ratio of serum LXA4 or RvD1 to PGD ↑ RvD1	[48,49]
***Cell culture studies***		
IOMT/RMS	↑ cell proliferation ↓ IL-6 secretion Inhibition of IL-1α, IL-1β, IL-2, IL-3, IL-6, and IL-16 secretion	[90]
Equibiaxial strain	↓ secretion of CCL18 ↓ cell proliferation ↓ secretions of MDC and IL-6	[93]
MFR/RMS	↓ apoptosis rate ↓ DAPK-2 ↑ CREBS133 ↑ FAK	[50]
ALDS/CSDS	↓ levels of IL-6 and IL-8	[100]
Static isotropic tensile strain, short-term high-frequency cyclic tension, dynamic tensile stretching,	*static tensile strain*: ↓ COL1A2 ↑ TNF-α,*COX-2, IL-6, IL-1ß* *short-term high-frequency cyclic tension*: ↑ *IL-6*, ↓ *IL-1ß* *dynamic tensile stretching*: ↓ COL1A2, TNF-α, IL-6, IL-1ß	[101]
Static progressive stretching/post-traumatic knee contracture model	↓ collagen proliferation	[59]
CTS/Il-1β-induced inflammation	Reversion of Il-1β-induced: iNOS and COX-2 expression NO and PGE2 synthesis MMP-9, MMP-13, MMP-1 gene expression ↑ collagen type II production, suppression of IL-1β-dependent collagenase synthesis Reversion of IL-1β-induced down TIMP2 Block of IL-1β-induced inhibition of type-I collagen synthesis inhibition of nuclear translocation of NF-κB ↓ IκBα and IκBβ transcription inhibition of IκBα and IκBβ degradation nuclear translocation of IκBα	[51,111,112,113]
DTF	↓ the mRNA expression of IL-1β inhibition of IL-1β-dependent induction of iNOS Inhibition of the expression of TNF-α and MMP-13	[115]
Static tissue stretch/injury-induced inflammation	ex vivo: ↓ TGF-β1, IL-6 in vivo: no increase in type-1 procollagen observed in stretched group after injury	[122]
Dynamic compressive strain/Il-1β-induced inflammation chondrocytes	↓ nitrate and PGe2 synthesis ↑ DNA synthesis (3H-thymidine incorporation) ↑ sulfate incorporation	[114]
CTS and gallic acid/osteoarthritic human articular chondrocytes	↑ glycosaminoglycan, collagen type II and IX	[121]

↑ increase/upregulation; ↓ decrease/downregulation.

## Data Availability

Not applicable.
